# A Bayesian Adaptive Design for Combination of Three Drugs in Cancer Phase I Clinical Trials

**DOI:** 10.3844/amjbsp.2016.1.11

**Published:** 2016-08-25

**Authors:** Mourad Tighiouart, Quanlin Li, Steven Piantadosi, Andre Rogatko

**Affiliations:** Samuel Oschin Comprehensive Cancer Institute, 8700 Beverly Blvd., Los Angeles, CA 90048, United States

**Keywords:** Cancer Phase I Trials, Maximum Tolerated Dose Surface, Escalation with Overdose Control, Drug Combination, Dose Limiting Toxicity, Continuous Dose

## Abstract

We describe a Bayesian adaptive design for early phase cancer trials of a combination of three agents. This is an extension of an earlier work by the authors by allowing all three agents to vary during the trial and by assigning different drug combinations to cohorts of three patients. The primary objective is to estimate the Maximum Tolerated Dose (MTD) surface in the three-dimensional Cartesian space. A class of linear models on the logit of the probability of Dose Limiting Toxicity (DLT) are used to describe the relationship between doses of the three drugs and the probability of DLT. Trial design proceeds using conditional escalation with overdose control, where at each stage of the trial, we seek a dose of one agent using the current posterior distribution of the MTD of this agent given the current doses of the other two agents. The MTD surface is estimated at the end of the trial as a function of Bayes estimates of the model parameters. Operating characteristics are evaluated with respect to trial safety and percent of dose recommendation at dose combination neighborhoods around the true MTD surface.

## Introduction

Early phase cancer trials are designed to study safety and tolerability of cytotoxic and biologic agents and recommend the Maximum Tolerated Dose (MTD) for use in future phase II studies. These trials enroll patients with late-stage cancer who became refractory to all standard and conventional therapy (Roberts *et al*., 2004) and the dose allocated to the next cohort of patients depends on the dose levels given to all previous patients and their Dose Limiting Toxicity (DLT) status.

It is well known that combining cytotoxic and biologic drugs lead to targeting various signaling pathways. This strategy helps in reducing tumor resistance to chemotherapy, an event that is experienced by a significant proportion of advanced stage cancer patients. The majority of phase I trials use drug combinations of several agents. However, most of them are designed to estimate the MTD of a single drug for fixed dose levels of the others. Such designs may recommend a single safe dose for the combination but it may not be the optimal combination with respect to treatment efficacy. A motivating example is a recent phase Ib trial combining the drugs momelotinib, capecitabine and oxaliplatin in patients with relapse or refractory metastatic pancreatic cancer. Three dose levels for momelotinib were selected and two dose levels for each capecitabine and oxaliplatin were pre-specified by the clinicians and the 3+3 algorithm was used with predetermined dose levels escalation. This approach is clearly inefficient since it may produce at most one MTD and this MTD may not be the optimal efficacious dose. In this manuscript, dose levels of two or more drugs are allowed to vary during the trial. The goal is then to determine a subset of dose combinations that will produce the same DLT rate.

Denote by *A_j_, j* = 1,…, *K* the *K* drugs under study and *S_i_* ϲ R*^+^* be the set of all possible doses of drug *A_j_*. Let *x* = (*x*_1_,…, *x_K_*) be a dose combination and *S* = *S*_1_×…× *S_K_*. Let:

(1.1) Prob(DLT∣dose=x)=F(x,ξ)

Be a dose-toxicity model with *F* a known link function and *ξ* ∈ R*^d^* an unknown parameter. By definition, the MTD is the set *Γ* of dose combinations *x* that produce the same DLT rate *θ*:

(1.2) Γ={x∈S:F(x,ξ)=θ}

The DLT rate *θ* depends on the seriousness of treatment related toxicities with typical values selected in the interval [0.2, 0.4]. A dose finding trial is a sequential dose allocation design used to estimate the set *Γ* efficiently while minimizing the number of patients exposed to highly toxic doses. Model based designs for estimating one or more than one MTD or have been proposed by many authors in the last decade (Thall *et al*., 2003; Wang and Ivanova, 2008; Yuan and Yin, 2008; Yin and Yuan, 2009a; 2009b; Braun and Wang, 2010; Wages *et al*., 2011a; 2011b; Shi and Yin, 2013; Tighiouart *et al*., 2014; Riviere *et al*., 2014; Mander and Sweeting, 2015; Tighiouart *et al*., 2016). Except for the methods in (Thall *et al*., 2003; Tighiouart *et al*., 2014; Tighiouart *et al*., 2016), these designs do not extend to the case of continuous dose levels and it is not clear how they perform when the number of dose combinations is high. In this article, we extend the design proposed in (Tighiouart *et al*., 2014) by exploring the safety and tolerability of three drugs with continuous dose levels. The algorithm in (Tighiouart *et al*., 2014) is further extended by enrolling cohorts of three patients receiving different dose combinations determined according to Escalation with Overdose Control (EWOC) criteria (Babb *et al*., 1998; Tighiouart *et al*., 2005; Tighiouart and Rogatko, 2010).

The manuscript is organized as follows. In section 2, we describe the Bayesian model and the adaptive trial design for assigning dose combination to each cohort. In section 3, we present the operating characteristics of the proposed method with respect to safety of the design and efficiency of the estimate of the MTD. Section 4 contains some final remarks and discussion of possible extensions.

## Materials and Methods

### Dose-Toxicity Model

Let *A*, *B* and *C* be cytotoxic agents and suppose that the doses of these agents are continuous and standardized to be in the interval [0, 1]. We consider the dose-toxicity model of the form:

(2.1) $$\text{Prob}(\delta =1|x,y,z)=F(\beta_{0}+\beta_{1}x+\beta_{2}y+\beta_{3}z+\eta \mathrm{xyz})$$

where, *δ* is the binary indicator of DLT, *δ* = 1 if a patient given the dose combination (*x*, *y*, *z*) has DLT within one cycle of therapy and *δ* = 0 otherwise, *x* is the dose level of agent *A*, *y* is the dose level of agent *B*, *z* is the dose level of agent *C* and *F* is a known cumulative distribution function.

We assume that the three drugs are synergistic so that *η* > 0. We further assume that that the probability of DLT increases with the dose of any one of the agents when the other two are held constant. A necessary and sufficient condition for this property to hold is to assume *β_k_* > 0, *k* = 1, 2, 3. The MTD is any dose combination (x*, y*, z*) such that:

(2.2) $$\text{Prob}(\delta =1|x^{*},y^{*},z^{*})=\theta .$$

It follows from (2.1) and (2.2) that the MTD is the set of dose combinations:

(2.3) $$\Gamma =\left\{(x^{*},y^{*},z^{*}):0\leq x^{*},y^{*},z^{*}\leq 1,z^{*}=\frac{F^{-1}(\theta )-\beta_{0}-\beta_{1}x^{*}-\beta_{2}y^{*}}{\beta_{3}+\eta x^{*}y^{*}}\right\}$$

We further reparameterize model (2.1) in terms of *Γ_A_*_|00_, the MTD of drug *A* when the level of drugs *B* and *C* are at their lowest available doses, *Γ_B_*_|00_, the MTD of drug *B* when the level of drugs *A* and *C* are at their lowest available doses, *Γ_C_*_|00_, the MTD of drug *C* when the level of drugs *A* and *B* are at their lowest available doses, *ρ*_0_, the probability of DLT at the minimum available doses of agents *A*, *B* and *C* corresponding to *x* = *y* = *z* = 0 and the interaction parameter *η*. This reparameterization is convenient to clinicians since prior information on *ρ*_0_, *Γ_A_*_|00_, *Γ_B_*_|00_ and *Γ_C_*_|00_ may be available from other trials. In this manuscript, we will assume that 0 ≤ *Γ_A_*_|00_, *Γ_B_*_|00_, *Γ_C_*_|00_ ≤ 1, i.e., the MTD of each agent when the other ones are held at their minimum available doses in the trial is within the range of available doses in the trial. It follows that:

(2.4) $$\left\{ \begin{array}{l} \beta_{0}=F^{-1}(\rho_{0})\\ \beta_{1}=(F^{-1}(\theta )-F^{-1}(\rho_{0}))/\Gamma_{A|00}\\ \beta_{2}=(F^{-1}(\theta )-F^{-1}(\rho_{0}))/\Gamma_{B|00}\\ \beta_{3}=(F^{-1}(\theta )-F^{-1}(\rho_{0}))/\Gamma_{C|00} \end{array}\right.$$

The MTD in (2.3) becomes:

(2.5) $$\Gamma =\left\{(x^{*},y^{*},z^{*}):0\leq x^{*},y^{*},z^{*}\leq 1,z^{*}=\frac{\left(F^{-1}(\theta )-F^{-1}(\rho_{0})\right)(1-x^{*}/\Gamma_{A|00}-y^{*}/\Gamma_{B|00})}{\left(\left(F^{-1}(\theta )-F^{-1}(\rho_{0})\right)/\Gamma_{C|00}\right)+\eta x^{*}y^{*}}\right\}$$

Let *D_n_* = {(*x_i_*, *y_i_*, *z_i_*, *δ_i_*), *i* = 1,…, *n*} be the data after enrolling *n* patients in the trial. Let *G*(*θ*, *ρ*_0_) = *F*^−1^(*θ*) − *F*^−1^(*ρ*_0_). The likelihood function for the model parameters is:

(2.6) $$L(\rho_{0},\Gamma_{A|00},\Gamma_{B|00},\Gamma_{C|00},\eta |D_{n})=\prod_{i=1}^{n}{\left(H(\rho_{0},\Gamma_{A|00},\Gamma_{B|00},\Gamma_{C|00},\eta ;x_{i},y_{i},z_{i})\right)}^{\delta_{i}}\times {\left(1-H(\rho_{0},\Gamma_{A|00},\Gamma_{B|00},\Gamma_{C|00},\eta ;x_{i},y_{i},z_{i})\right)}^{1-\delta_{i}}$$

where:

(2.7) $$H(\rho_{0},\Gamma_{A|00},\Gamma_{B|00},\Gamma_{C|00},\eta ;x_{i},y_{i},z_{i})=F\left(F^{-1}(\rho_{0})+\frac{G(\theta ,\rho_{0})}{\Gamma_{A|00}}x_{i}+\frac{G(\theta ,\rho_{0})}{\Gamma_{B|00}}y_{i}+\frac{G(\theta ,\rho_{0})}{\Gamma_{C|00}}z_{i}+\eta x_{i}y_{i}z_{i}\right)$$

### Prior and Posterior Distributions

Equation 2.4 implies that 0 <*ρ*_0_<*θ* since *β_k_*> 0, *k* =1,2,3. We assume that *ρ*_0_, *Γ_A_*_|00_, *Γ_B_*_|00_, *Γ_A_*_|00_ and *η* are independent *a priori* with *ρ*_0_/*θ* ~beta(*a*_0_, *b*_0_), *Γ_A_*_|00_ ~ beta(*a*_1_, *b*_1_), *Γ_B_*_|00_ ~ beta(*a*_2_, *b*_2_), *Γ_C_*_|00_ ~ beta(*a*_3_, *b*_3_) and *η* ~ gamma(*a*, *b*) with mean *E*(*η*) = *a* / *b* and variance *Var*(*η*) = *a* / *b*^2^. Under lack of prior information about the probability of DLT at the minimum dose combination (0,0, 0) and the MTDs of agents *A*, *B* and *C* when used as single agents, we take *a_k_* = *b_k_* = 1, *k* = 0, 1, 2, 3 which corresponds to a uniform prior for *ρ*_0_ in [0, *θ*] and uniform priors for the parameters *Γ_A_*_|00_, *Γ_B_*_|00_, *Γ_C_*_|00_ in [0, 1].

Following the work in (Tighiouart *et al*., 2014), we specify a diffuse prior distribution on the interaction coefficient *η* as follows. To select the prior mean for *η*, substitute the prior mean values of *ρ*_0_, *Γ_A_*_|00_, *Γ_B_*_|00,_ *Γ_C_*_|00_ in place of these parameters in (2.5) and consider the MTD surface passing through the four points with coordinates (0, *E*(*Γ_B_*_|00_), 0), (*E*(*Γ_A_*_|00_), 0, 0), (0, 0, *E*(*Γ_C_*_|00_)) and (*E*(*Γ_A_*_|00_)/6, *E*(*Γ_B_*_|00_)/6, *E*(*Γ_C_*_|00_)/6). The prior mean of *η* is solution to the equation:

(2.8) $$E(\Gamma_{C|00})/6=\frac{G(\theta ,E(\rho_{0}))\left(1-\left(E(\Gamma_{A|00})/6\right)/E(\Gamma_{A|00})-\left(E(\Gamma_{B|00})/6\right)/E(\Gamma_{B|00})\right)}{\left(G(\theta ,E(\rho_{0}))/E(\Gamma_{C|00})\right)+E(\eta )E(\Gamma_{A|00})E(\Gamma_{B|00})/36}$$

It follows that:

(2.9) $$E(\eta )=\frac{108\left(F^{-1}(\theta )-F^{-1}(E(\rho_{0}))\right)}{E(\Gamma_{A|00})E(\Gamma_{B|00})E(\Gamma_{C|00})}$$

**Fig. 1:**
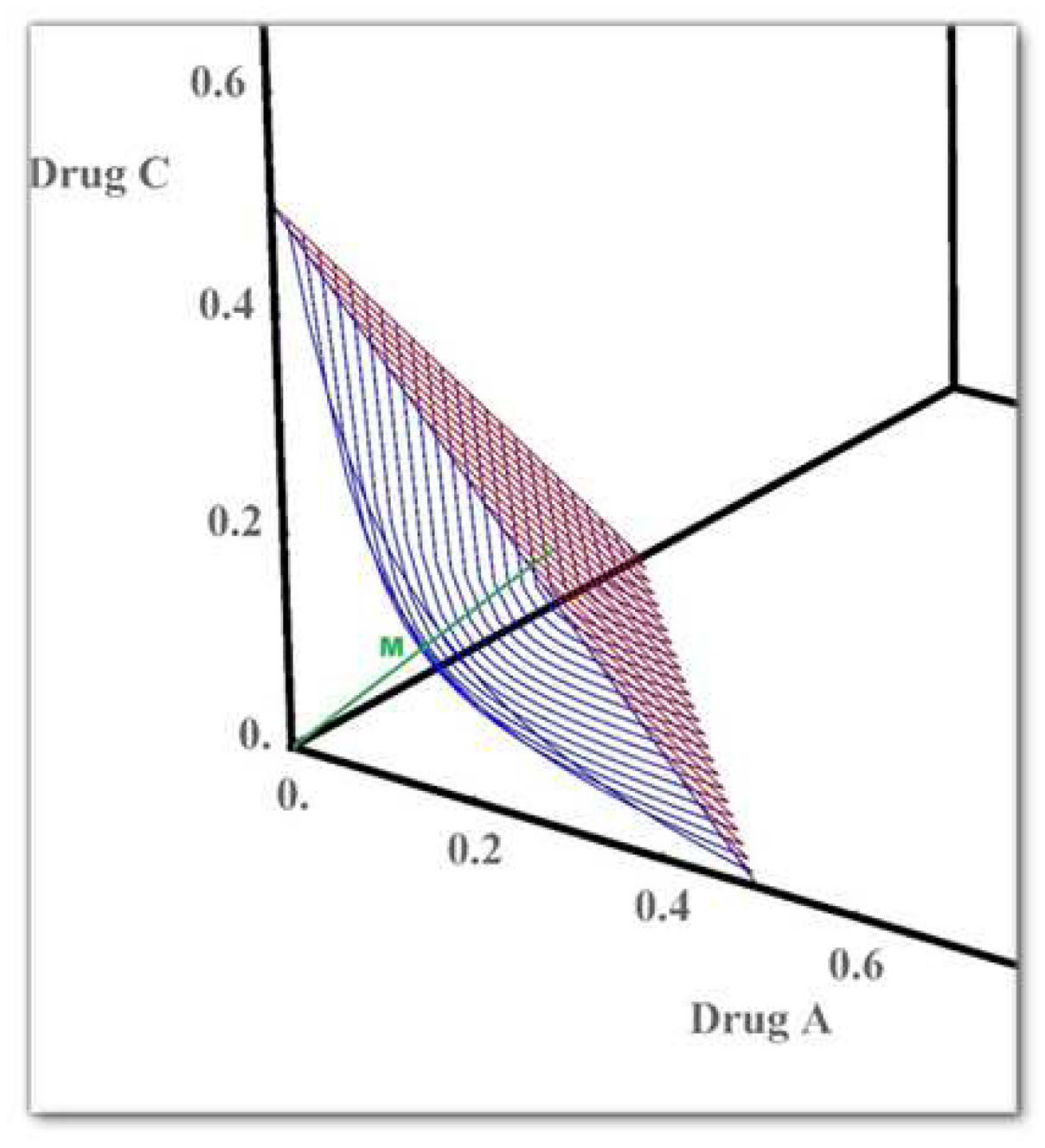
MTD surface when the interaction parameter *η* = 0 (red) and when the interaction parameter *η* = 790 (blue). The green line stretches from the minimum dose combination (0, 0, 0) to the centroid of the MTD surface shown in red

The idea here is to draw the MTD surface *Γ*_0_ passing through the points of average MTDs (*E*(*Γ_A_*_|00_), 0, 0), (0, *E*(*Γ_B_*_|00_), 0) and (0, 0, *E*(*Γ_C_*_|00_)) when the interaction coefficient is 0 (see the red surface in Fig. 1). Then, draw a line passing through (0, 0, 0) and the centroid of the MTD surface *Γ*_0_ (see the green line in Fig. 1). MTD surfaces passing through the points of average MTDs (*E*(*Γ_A_*_|00_), 0, 0), (0, *E*(*Γ_B_*_|00_), 0) and (0, 0, *E*(*Γ_C_*_|00_)) will cross the green line as *η* increases. Among these surfaces, we select the one passing through the midpoint M of the green line with coordinates (*E*(*Γ_A_*_|00_)/6, *E*(*Γ_B_*_|00_)/6, *E*(*Γ_C_*_|00_)/6). This surface is shown in blue in Fig. 1. The value of *η* corresponding to this MTD surface is found by solving Equation 2.8 and the solution is selected as the prior expected value for *η* and is given by (2.9). A large variance is selected for this prior.

Using Bayes rule, the posterior distribution of the model parameters is proportional to the product of the likelihood and prior distribution:

(2.10) $$\begin{array}{l} \pi (\rho_{0},\Gamma_{A|00},\Gamma_{B|00},\Gamma_{C|00},\eta |D_{n})\\ \propto \prod_{i=1}^{n}{\left(H(\rho_{0},\Gamma_{A|00},\Gamma_{B|00},\Gamma_{C|00},\eta ;x_{i},y_{i},z_{i})\right)}^{\delta_{i}}\\ \times {\left(1-H(\rho_{0},\Gamma_{A|00},\Gamma_{B|00},\Gamma_{C|00},\eta ;x_{i},y_{i},z_{i})\right)}^{1-\delta_{i}}\\ \eta^{a-1}e^{-b\eta }{\rho_{0}}^{a_{0}-1}{\left(1-\rho_{0}\right)}^{b_{0}-1}\\ \times {\left(\Gamma_{A|00}\right)}^{a_{1}-1}{\left(1-\Gamma_{A|00}\right)}^{b_{1}-1}{\left(\Gamma_{B|00}\right)}^{a_{2}-1}\\ {\left(1-\Gamma_{B|00}\right)}^{a_{2}-1}{\left(\Gamma_{C|00}\right)}^{a_{3}-1}{\left(1-\Gamma_{C|00}\right)}^{a_{3}-1} \end{array}$$

The software Win BUGS (Lunn *et al*., 2000) and JAGS were used to estimate features of the posterior distribution of these parameters and estimate the operating characteristics of the adaptive design described below.

### Trial Design

Dose escalation or de-escalation is designed by treating successive cohorts of three patients. For each cohort, each patient receives a dose of one agent determined using EWOC while holding the other two agents constant. For example, if agents *A* and *B* are held constant at levels *x* and *y*, respectively, the dose of agent *C* is *z* such that the posterior probability that *z* exceeds the MTD of agent *C* given *A* = *x* and *B* = *y* equals to a feasibility bound *α*. The algorithm proceeds as follows:

1. Each patient in the first cohort of three patients receives the same dose combination (*x*_1_,*y*_1_, *z*_1_) = (0, 0, 0). Let *D*_3_ = {(*x*_1_, *y*_1_, *z*_1_, *δ*_1_), (*x*_2_, *y*_2_, *z*_2_, *δ*_2_), (*x*_3_, *y*_3_, *z*_3_, *δ*_3_)}
2. In the second cohort of three patients, patient 4 receives dose (*x*_4_, *y*_1_, *z*_1_), patient 5 receives dose (*x*_2_, *y*_5_, *z*_2_), patient 6 receives dose (*x*_3_, *y*_3_, *z*_6_), where *x*_4_ is the *α*-th percentile of *π*(*Γ*_*A*|*B*=*y*_1__, _*C*=*z*_1__|*D*_3_), the posterior distribution of the MTD of drug *A* given that *B* = *y*_1_, *C* = *z*_1_, *y*_5_ is the *α*-th percentile of *π*(*Γ*_*B*|*A*=*x*_1_*C*=*z*_1__| *D*_3_) and *z*_6_ is the *α*-th percentile of *π*(*Γ*_*C*|*A*=*x*_1__, _*B*=*y*_1__| *D*_3_). These posterior distributions are easily obtained from the MCMC output since *Γ_A_*_|_*_B_*_=_ *_y_*_,_ *_C = z_*, *Γ_B_*_|_*_A_*_=_ *_x_*_,_ *_C = z_* and *Γ_C_*_|_*_A_*_=_ *_x_*_,_ *_B = y_* can be expressed explicitly in terms of the model parameters *ρ*_0_, *Γ_A_*_|00_, *Γ_B_*_|00,_ *Γ_C_*_|00_ and *η*
3. In the *i*-th cohort of three patients, patient 3*i* -2 receives dose (*x*_3_*_i_*_-2_, *y*_3_*_i_*_-4_, *z*_3_*_i_*_-3_), patient 3*i* -1 receives dose (*x*_3_*_i_*_-5_, *y*_3_*_i_*_-1_, *z*_3_*_i_*_-3_), patient 3*i* receives dose (*x*_3_*_i_*_-5_, *y*_3_*_i_*_-4_, *z*_3_*_i_*), where $x_{3i-2}=\Pi_{\Gamma_{A|B=y_{3i-4},C=z_{3i-3}}}^{-1}\left(\alpha |D_{3i-3}\right),y_{3i-1}=\Pi_{\Gamma_{B|A=x_{3i-5},C=z_{3i-3}}}^{-1}\left(\alpha |D_{3i-3}\right)$ and $z_{3i}=\Pi_{\Gamma_{C|A=x_{3i-5},B=y_{3i-4}}}^{-1}\left(\alpha |D_{3i-3}\right)$. Here, $\Pi_{\Gamma_{A|B=y,C=z}}^{-1}\left(\alpha |D_{i}\right)$ denotes the inverse cdf of the posterior distribution *π*(*Γ_A_*_|_*_B_*_=_*_y_*_,_ *_C_*_=_*_z_*| *D_i_*)
4. Repeat step 3 until n patients are enrolled to the trial or the following stopping rule holds

### Stopping Rule

Since 0 < *ρ*_0_< *θ*, the posterior probability of DLT at the minimum dose combination is always bounded by the target probability of DLT *θ*. Therefore, *ad hoc* stopping rules are necessary for trial conduct and are discussed with the clinician. For example, a decision to suspend accrual to the trial and revise the dose range available in the trial may be made if 2 or 3 DLTs are encountered in the first cohort of patients treated at the minimum dose combination (0, 0, 0).

At the conclusion of the trial, the MTD surface is estimated using (2.5) as:

(2.11) $$\hat{\Gamma }=\left\{(x^{*},y^{*},z^{*}):0\leq x^{*},y^{*},z^{*}\leq 1,z^{*}=\frac{\left(F^{-1}(\theta )-F^{-1}({\hat{\rho }}_{0})\right)(1-x^{*}/{\hat{\Gamma }}_{A|00}-y^{*}/{\hat{\Gamma }}_{B|00})}{\left(\left(F^{-1}(\theta )-F^{-1}({\hat{\rho }}_{0})\right)/{\hat{\Gamma }}_{C|00}\right)+\hat{\eta }x^{*}y^{*}}\right\}$$

where, *ρ̂*_0_, *Γ̂_A_*_|00_, *Γ̂_B_*_|00_, *Γ̂_C_*_|00_, *η̂* are the posterior medians given the data *D_n_*.

## Simulation Studies

### Simulation Set Up and Scenarios

We evaluate the performance of this method by deriving the operating characteristics assuming a logistic link function *F*(*u*) = (1 + *e*^−^*^u^*)^−1^ for the true and working model. Operating characteristics under model misspecification will be investigated in future work. The target probability of DLT is fixed at *θ* = 0.33 and the trial sample size is *n* = 60 patients. We considered 8 scenarios for the true MTD surface and the corresponding parameters (*ρ*_0_, *Γ_A_*_|00_, *Γ_B_*_|00_, *Γ_C_*_|00_, *η*) are found in Table 1. These scenarios reflect different locations for the true MTD surface in the Cartesian space with varying distances from the minimum dose combination.

We used uniform priors for *ρ*_0_, *Γ_A_*_|00_, *Γ_B_*_|00_, *Γ_C_*_|00_ to reflect a lack of prior knowledge about the toxicity profiles of the three agents and using (2.9), the prior mean for *η* is *E*(*η*) = 790. We took a large prior variance for *η*, *Var*(*η*) = 790 and for each scenario, we simulated *m* = 1000 trials. In all simulations, we used a single MCMC chain to summarize posterior estimates after discarding the first 5000 samples and another 5000 updates to estimate features of the posterior distributions of the model parameters. No thinning of the MCMC chains were used and convergence was assessed using the package CODA in R.

### Design Operating Characteristics

For each scenario, we present an estimate of the MTD surface:

(3.1) $$\bar{\Gamma }=\left\{(x^{*},y^{*},z^{*}):0\leq x^{*},y^{*},z^{*}\leq 1,z^{*}=\frac{\left(F^{-1}(\theta )-F^{-1}({\bar{\rho }}_{0})\right)(1-x^{*}/{\bar{\Gamma }}_{A|00}-y^{*}/{\bar{\Gamma }}_{B|00})}{\left(\left(F^{-1}(\theta )-F^{-1}({\bar{\rho }}_{0})\right)/{\bar{\Gamma }}_{C|00}\right)+\bar{\eta }x^{*}y^{*}}\right\}$$

where, *F*(·) is the logistic function and *ρ̄*_0_, *Γ̄_A_*_|00_, *Γ̄_B_*_|00_, *Γ̄_C_*_|00_, *η̄* are the average posterior medians of the parameters *ρ*_0_, *Γ_A_*_|00_, *Γ_B_*_|00_, *Γ_C_*_|00_, *η* from all *m* = 1000 trials. For trial efficiency, we present the pointwise average relative minimum distance from the true MTD surface to the estimated MTD surface *d*_(_*_x_*_,_ *_y_*_,_ *_z_*_)_ as described in (Tighiouart *et al*., 2014; 2016) for two drugs. For *l* = 1,…,*m*, let *Γ_l_* be the estimated MTD surface and let *Γ_true_* be the true MTD surface. For every point (*x*, *y*, *z*) ∈ *Γ_true_*, let:

(3.2) $$d_{\left(x,y,z\right)}^{\left(l\right)}=\mathrm{sign}(z^{\prime }-z)\times \min_{\left\{(x^{*},y^{*},z^{*}):(x^{*},y^{*},z^{*})\in \Gamma_{l}\right\}}{\left({\left(x-x^{*}\right)}^{2}+{\left(y-y^{*}\right)}^{2}+{\left(z-z^{*}\right)}^{2}\right)}^{1/2}$$

where, *z′* is such that (*x*, *y*, *z′*) ∈ *Γ_l_*. This represents the minimum relative distance of the point (*x*, *y*, *z*) on the true MTD surface to the estimated MTD surface *Γ_l_*. If the point (*x*, *y*, *z*) is below *Γ_l_*, then $d_{\left(x,y,z\right)}^{\left(l\right)}$ is positive. Otherwise, it is negative. Let:

(3.3) $$d_{\left(x,y,z\right)}=m^{-1}\sum_{l=1}^{m}d_{\left(x,y,z\right)}^{\left(l\right)}$$

This is the pointwise average relative minimum distance from the true MTD surface to the estimated MTD surface which can be interpreted as the pointwise average bias in estimating the MTD. The last measure of efficiency we consider is:

(3.4) $$m^{-1}\sum_{l=1}^{m}I\left(|d_{\left(x,y,z\right)}^{\left(l\right)}|\;\leq p\Delta (x,y,z)\right)$$

where, Δ(*x*, *y*, *z*) = (*x*^2^ + *y*^2^ + *z*^2^)^0.5^.This is the point wise percent of trials for which the minimum distance of the point (*x*, *y*, *z*) on the true MTD surface to the estimated MTD surface *Γ_i_* is no more than (100×*p*)% of the true MTD. This can be interpreted as the point wise percent of MTD recommendation for a given tolerance *p*.

## Results

### Trial Safety

Table 1 shows that the average percent of DLTs varies between 16 and 33% across all 8 scenarios. This average DLT rate tends to be lower when the true MTD curve is farther away from the minimum dose combination, consistent with the results drug combinations with two agents (Tighiouart *et al*., 2014). Table 1 also shows that the percent of trials with an excessive number of DLTs as defined by a DLT rate exceeding *θ* + 0.1 is essentially 0. Based on these results under these scenarios, we conclude that the trial design is safe.

### Trial Efficiency

**Fig. 2:**
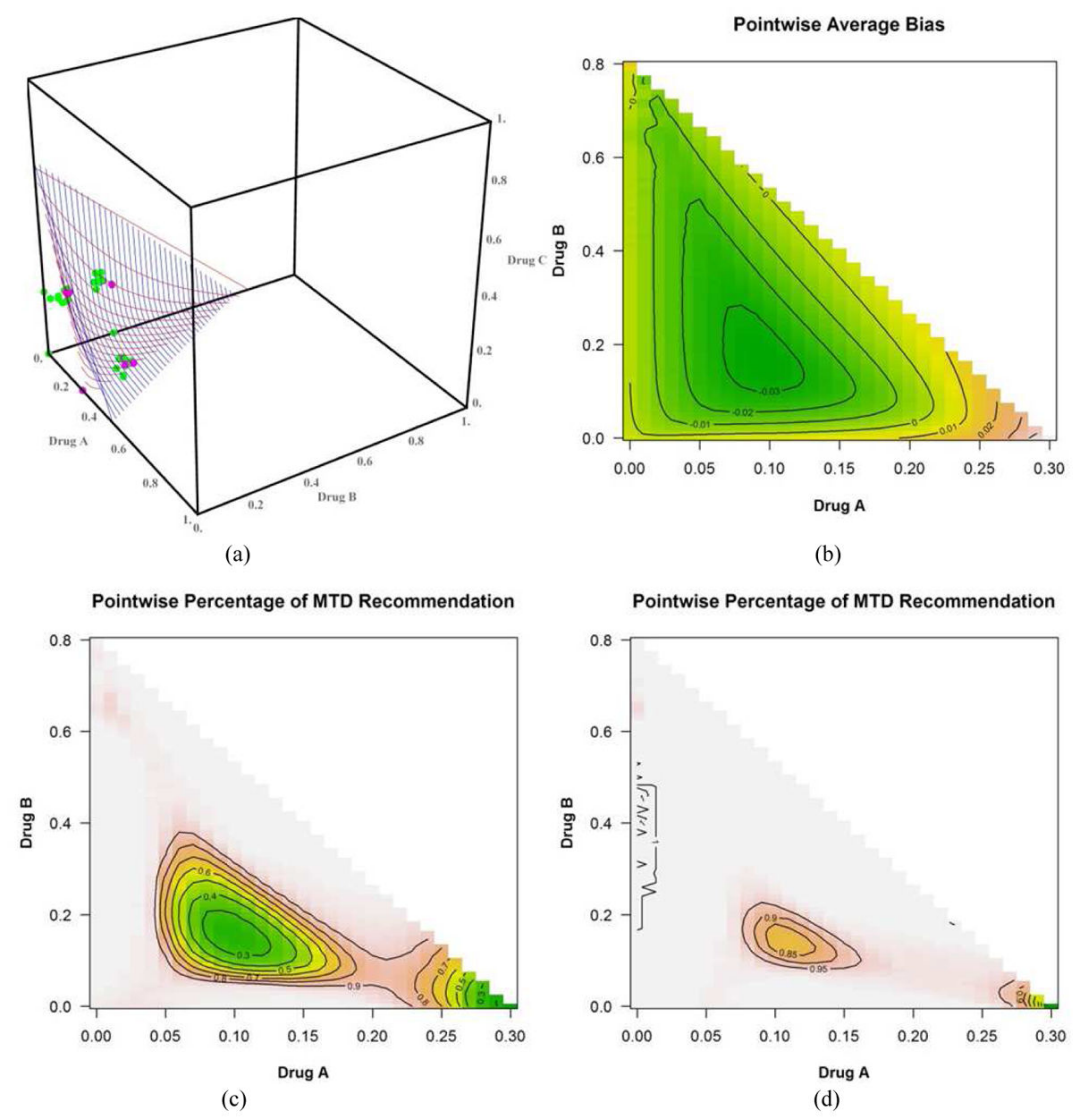
(a) True (in red) and estimated (in blue) MTD surface. The dots represent the dose combinations from the last simulated trial, green indicating no DLT and pink DLT, (b) dose combination contours for selected values of the pointwise average bias, (c) dose combination contours for selected values of the pointwise percent selection with tolerance *p* = 0.1 and (d) with tolerance *p* = 0.2

**Fig. 3:**
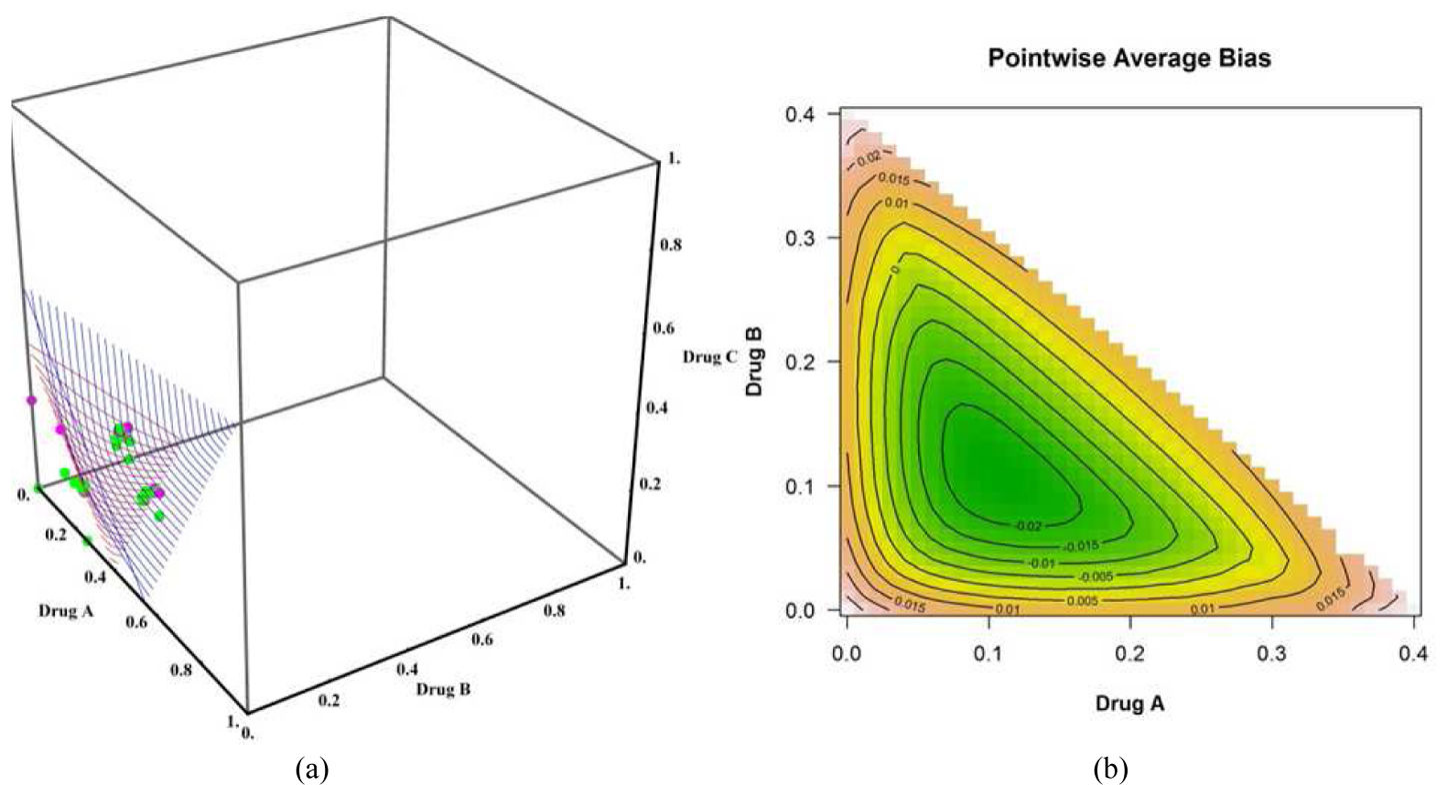

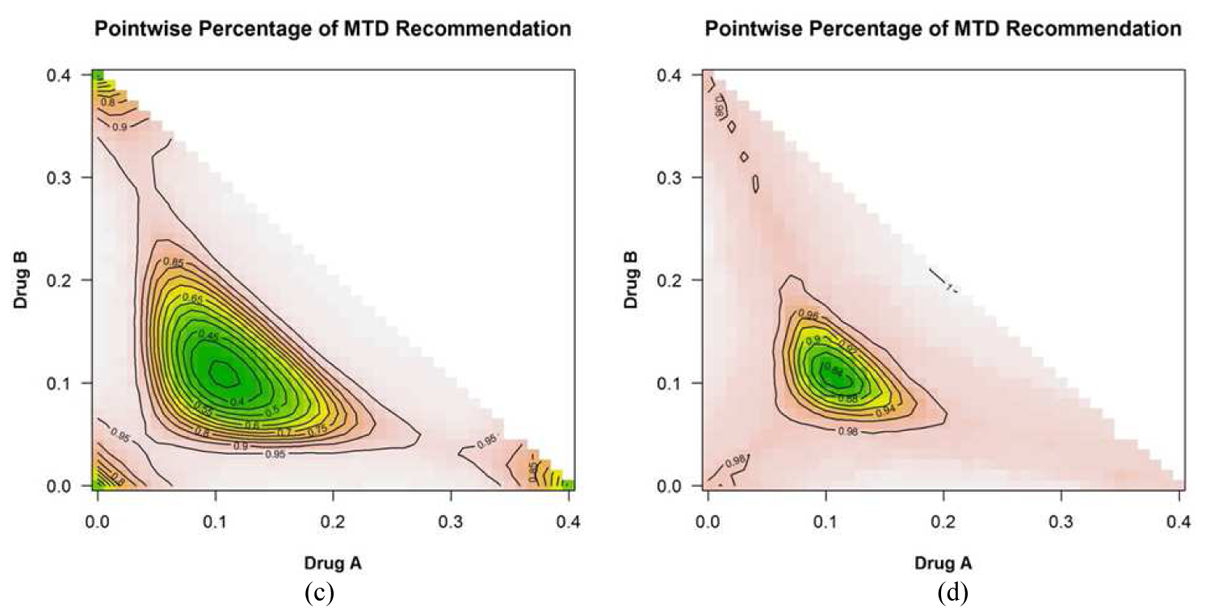
(a) True (in red) and estimated (in blue) MTD surface. The dots represent the dose combinations from the last simulated trial, green indicating no DLT and pink DLT, (b) dose combination contours for selected values of the pointwise average bias, (c) dose combination contours for selected values of the pointwise percent selection with tolerance *p* = 0.1 and (d) with tolerance *p* = 0.2

**Fig. 4:**
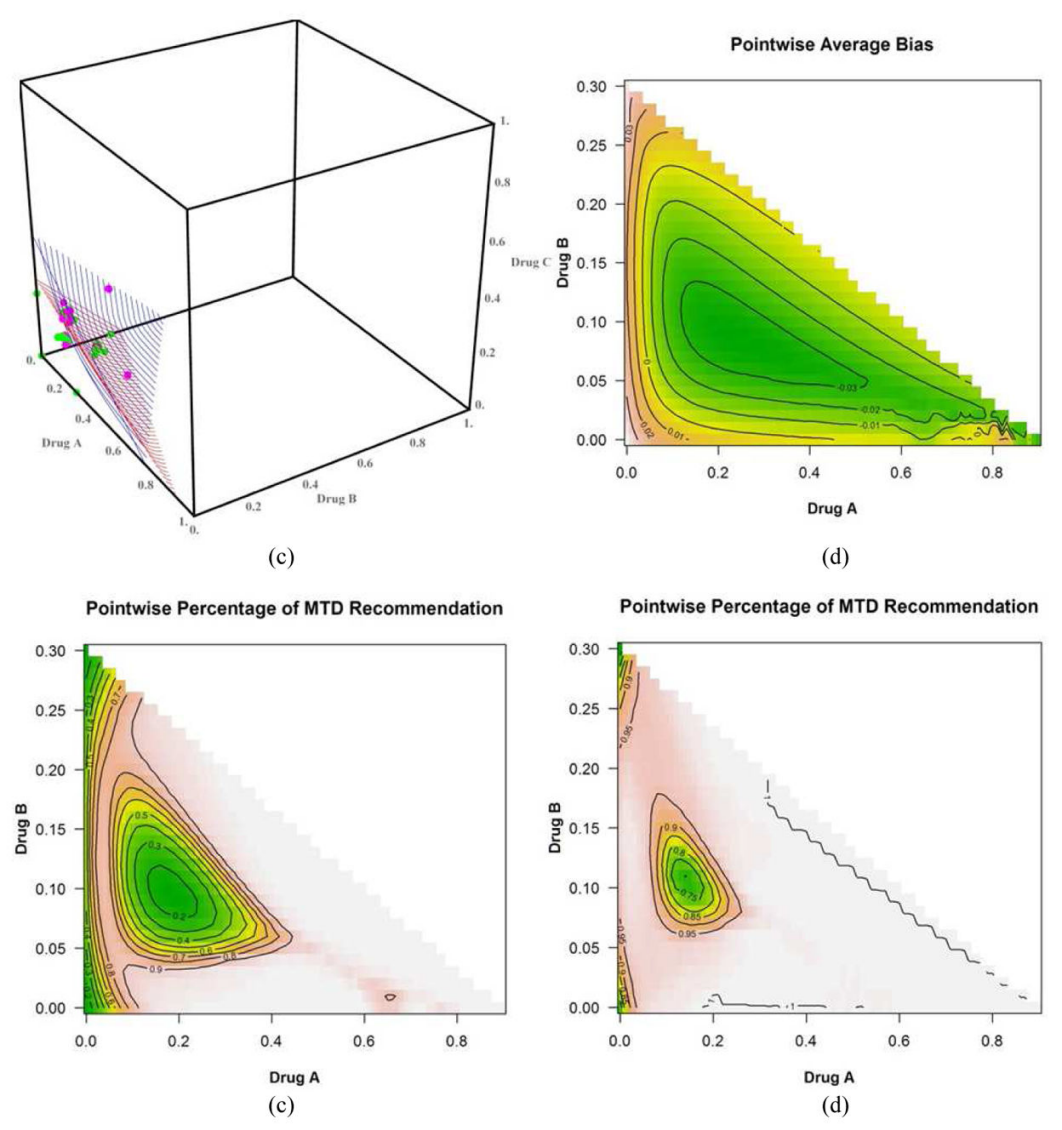
(a) True (in red) and estimated (in blue) MTD surface. The dots represent the dose combinations from the last simulated trial, green indicating no DLT and pink DLT, (b) dose combination contours for selected values of the pointwise average bias, (c) dose combination contours for selected values of the pointwise percent selection with tolerance *p* = 0.1 and (d) with tolerance *p* = 0.2

**Fig. 5:**
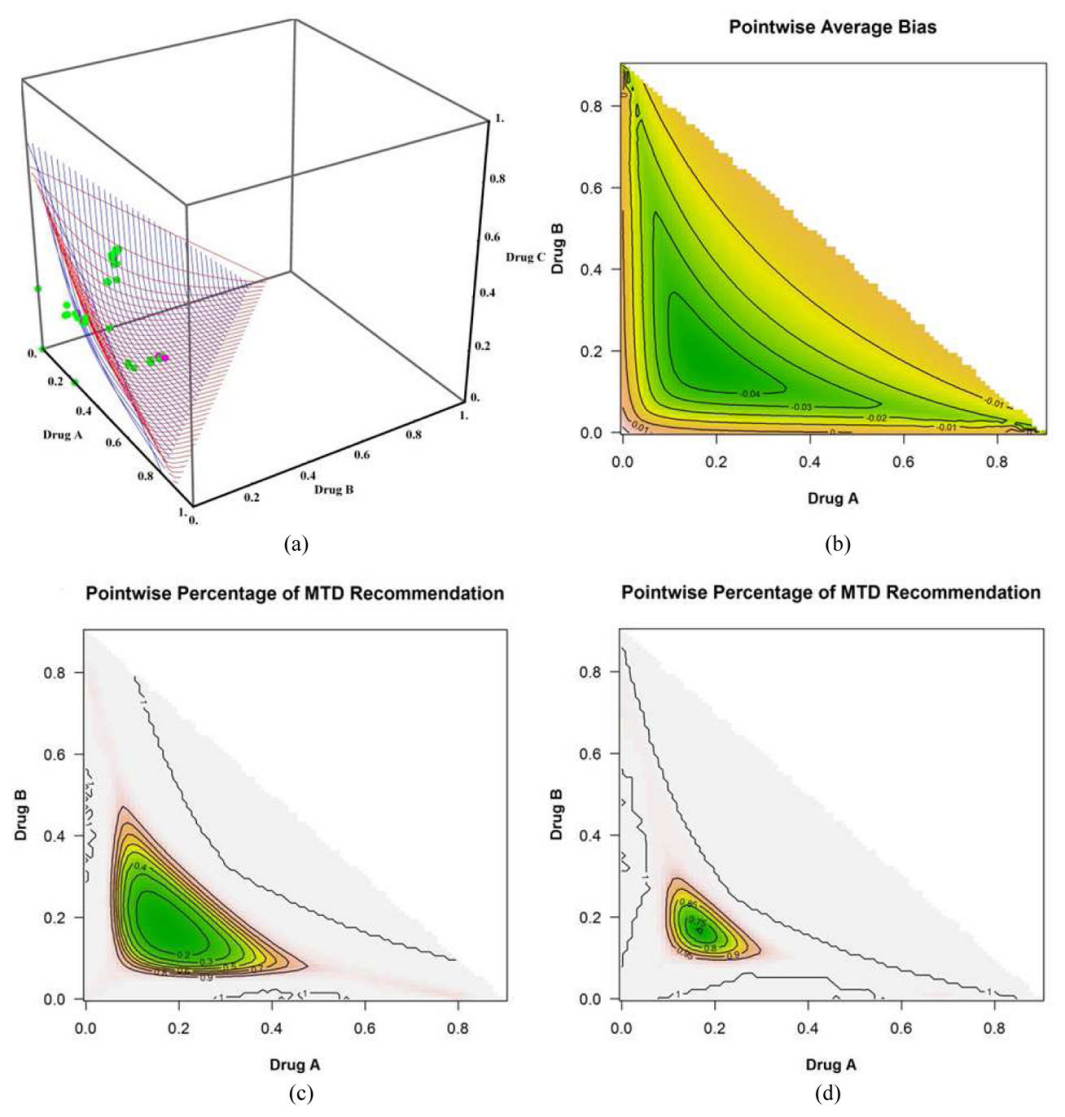
(a) True (in red) and estimated (in blue) MTD surface. The dots represent the dose combinations from the last simulated trial, green indicating no DLT and pink DLT, (b) dose combination contours for selected values of the pointwise average bias, (c) dose combination contours for selected values of the pointwise percent selection with tolerance *p* = 0.1 and (d) with tolerance *p* = 0.2

**Fig. 6:**
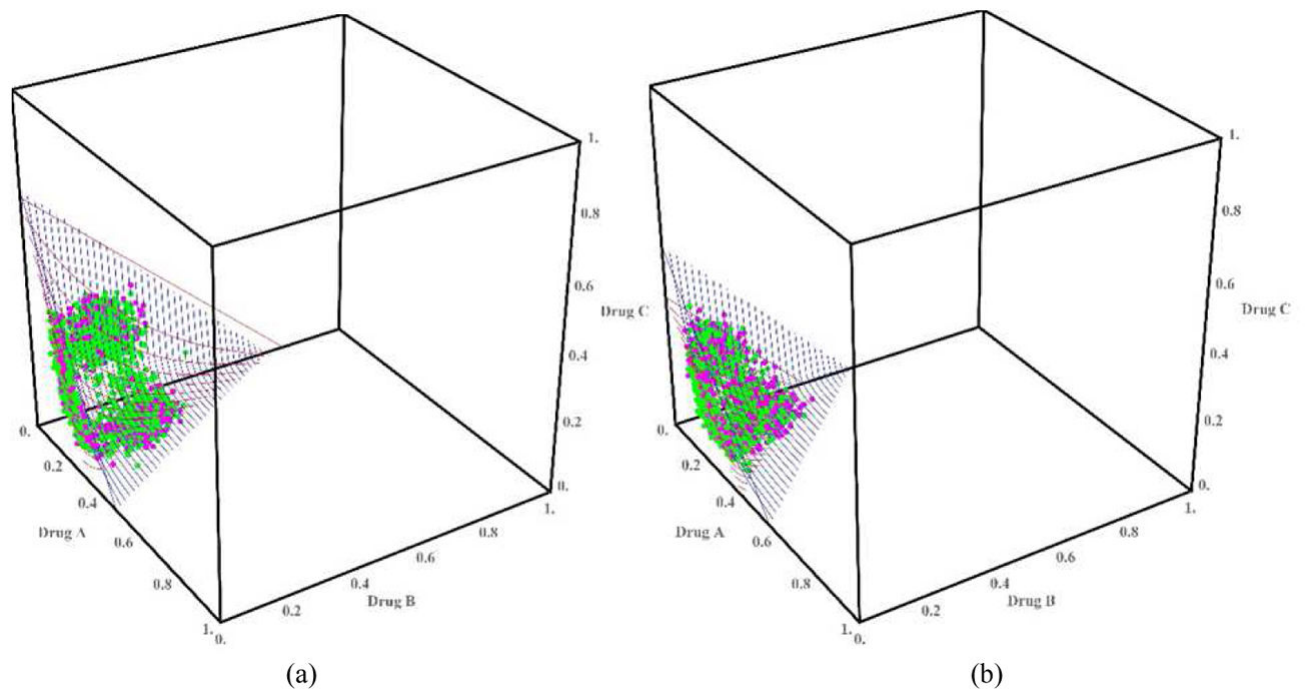

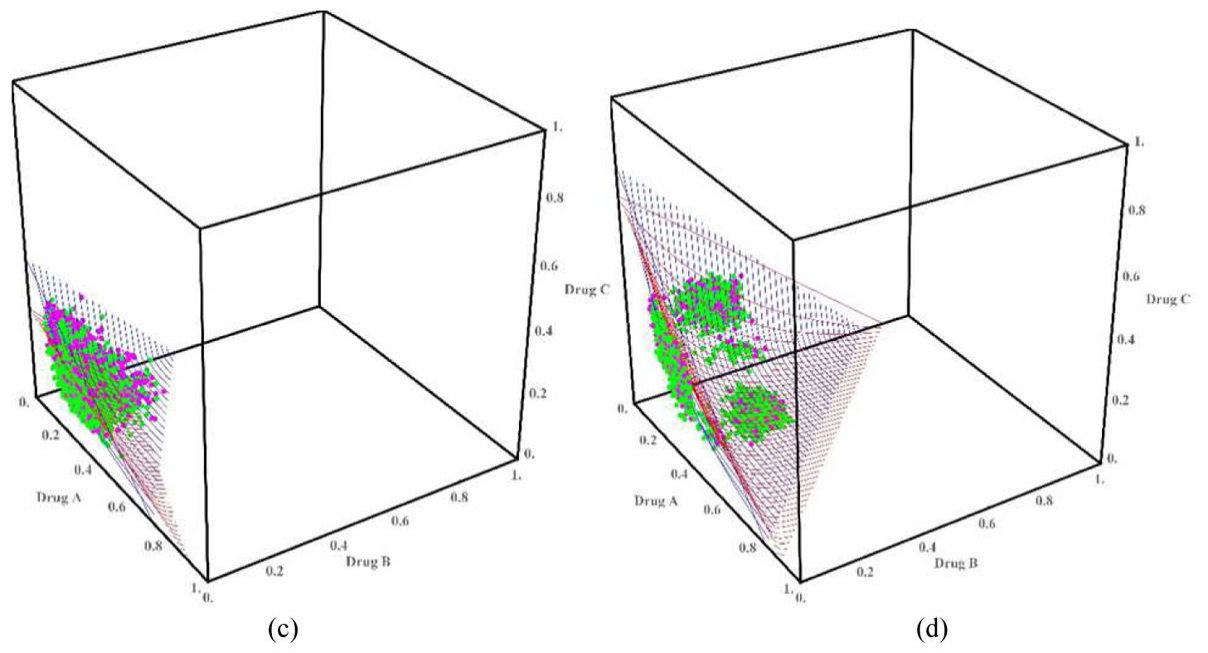
True (in red) and estimated (in blue) MTD surface for scenarios 1–4. The dots represent the dose combinations from the last cohort of three patients from all *m* = 1000 simulated trial, green indicating no DLT and pink DLT

**Fig. 7:**
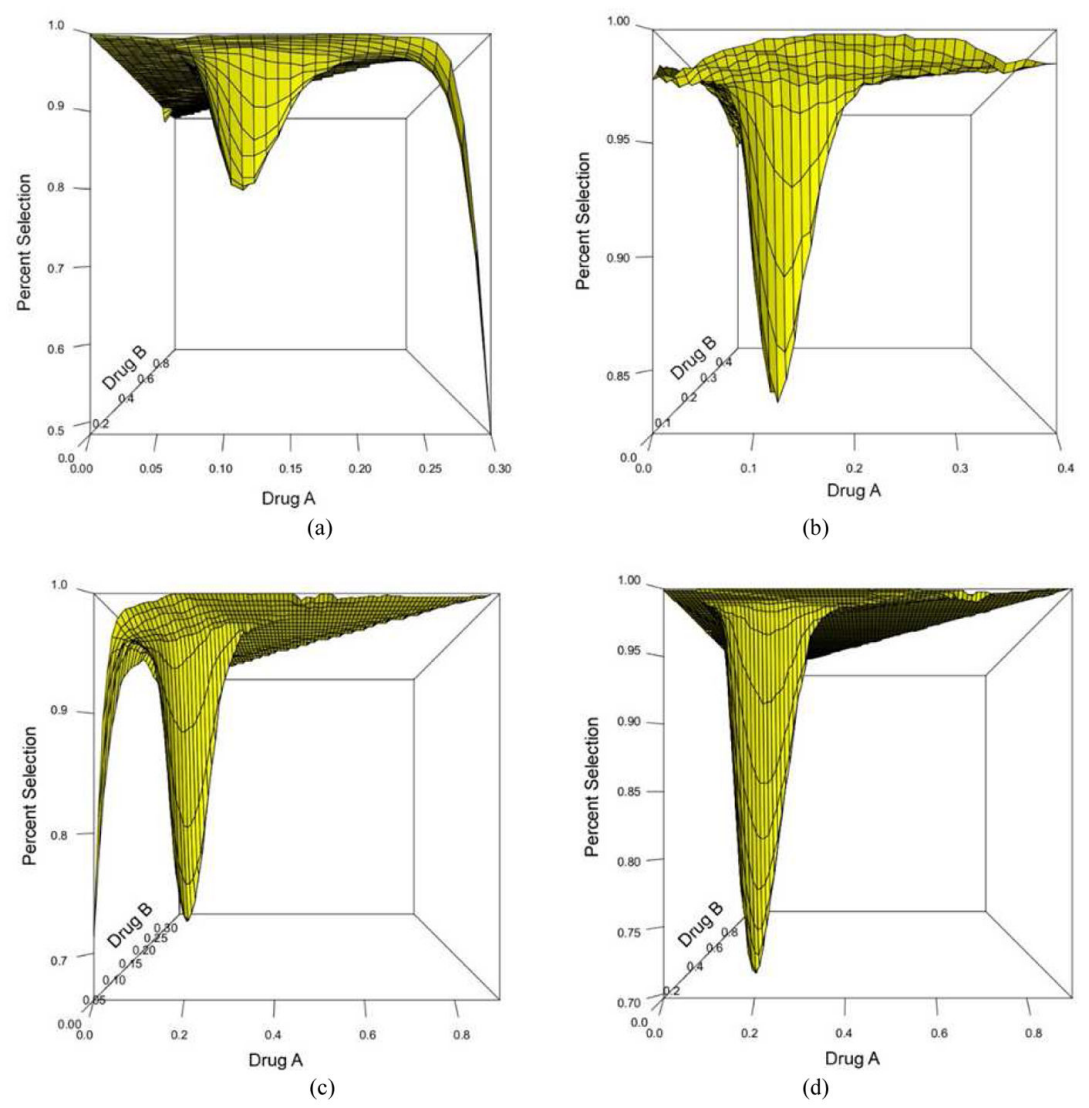
Three dimensional plots of the pointwise percent selection using a tolerance *p* = 0.2 for scenario 1 (top left), scenario 2 (top right), scenario 3 (bottom left) and scenario 4 (bottom right)

We discuss only the first 4 scenarios due to space and manuscript length considerations. Figure 2a shows the plot of the true(red) and estimated (blue) MTD surface under scenario 1 as described by its true parameter values in Table 1. The estimated MTD surface was obtained using (3.1). We can see that the estimated MTD surface is close to the true MTD surface except at the edge along drug *A*. Figure 2b shows the contours of the average bias varying from −0.03 to 0.04 for all combinations (*x*, *y*) such that (*x*, *y*, *z*) belongs to the true MTD surface. This shows that the average bias is negligible throughout all dose combinations on the MTD surface and that the average bias tends to increase as we approach dose combination (0.3, 0, 0). Figure 2a and c shows the contours of the pointwise percent selection for tolerances *p* = 0.1, 0.2. Using a tolerance of *p* = 0.1, the percent selection tends to be low around the middle part of the true MTD surface (30%) and increases as we move away from the center but decreases again around the edge defined by dose combination (0.3, 0, 0). When using the tolerance *p* = 0.2, the percent selection is very high across all the dose combinations on the true MTD surface (80% or higher). This can also be seen from the 3-dimensional plots of the pointwise percent selection under the four scenarios in Fig. 7 with percent selection varying between 70 and 100% for essentially all dose combinations. Similar conclusions can be made for scenarios 2, 3 and 4 shown in Fig. 3–5. These scenarios also show that the pointwise average bias is higher at the edges of the surface when the true MTD surface is close to the minimum dose combination (0, 0, 0). Figure 6 shows the true and estimated MTD surface under scenarios 1–4 along with the last dose combinations assigned to the last cohort of 3 patients from all 1000 simulated trials. After viewing these graphs from several angles, we found that the last dose combinations tend to cluster around the true MTD curve. Based on these results and others from scenarios not shown here, we conclude that the design is practically safe and relatively efficient in general in recommending the MTD surface estimate using the tolerance *p* = 0.1.

## Conclusion

The purpose of this manuscript was to extend the two-drug combination early phase trial design of Tighiouart *et al*. (2014) by using three agents and treating successive cohorts of three patients with different combinations for better exploration of the dose combination space. We showed that this is feasible using sample size of *n* = 60 patients under some scenarios for the true MTD surface. To the best of our knowledge, this is the first method geared towards estimating the MTD surface of three drugs based on continuous dose levels of the agents under consideration. The sample size used in our simulations is arbitrary and it corresponds to the previously used sample size of *n* = 40 for drug combinations of two agents (Tighiouart *et al*., 2014; 2016). Operating characteristics using a smaller sample size will be investigated in future work. We note that this sample size is conservative compared *n* = 60 for drug combinations of two agents studied in (Yin and Yuan, 2009a; 2009b). We also note that the priors used are vague and in practice, prior information about each agent can be used to calibrate the priors on *ρ*_0_, *Γ_A_*_|00_, *Γ_B_*_|00,_ *Γ_C_*_|00_ and similar operating characteristics may be obtained with a smaller sample size. The use of continuous dose levels is very common in clinical oncology research (Boodman, 2010). The method we presented is model based and the design alternates the use of single agent EWOC conditional on the dose level of the other agents. The dose-toxicity model we used only includes a three-way interaction term since including two-way interactions will result in three extra parameters. We plan to study the operating characteristics of this method under model misspecification where the DLT responses are generated from a class of models that include all two-way interaction terms in addition to the three-way interaction term. Prior information about toxicity data from each drug when used as single agents can be easily accounted for in the model but it is not required otherwise. We used vague priors for these parameters and proposed an ad hoc method to place a weakly informative prior distribution on the interaction term between the three drugs.

The assumption that the MTDs *Γ_A|_*_00_, *Γ_B|_*_00_ and *Γ_C|_*_00_are within the range of doses available in the trial may be too restrictive, especially if agents any of these agents were never used as single agents in human phase I trials. We plan to relax this condition using alternative model reparameterizations as in Tighiouart *et al*. (2016) in our future work. We also plan to study the performance of the proposed design when the true model does not belong to the class of dose-toxicity models in (2.1). Finally, we plan to assess the performance of the method when the doses of the three agents are discretized as in (Tighiouart *et al*., 2016) and compare it to the method of (Yin and Yuan, 2009b) after modifying the last step of the algorithm to allow estimation of more than one MTD.

Since the proposed method gives an estimated MTD surface, innovative phase II designs are needed to identify dose combinations on this surface with desirable level of efficacy. This can be established by constructive an adaptive design treating consecutive small cohorts of patients and update the efficacy surface as the treatment response is resolved. Such approach is under work in two-drug combinations and some preliminary results can be found in (Tighiouart *et al*., 2015).

## Figures and Tables

**Table 1:** Operating characteristics summarizing trial safety

Scenario (*ρ*_0_, *Γ_A_*_|00_, *Γ_B_*_|00_, *Γ_C_*_|00_, *η*)	Average	% Trials: DLT rate	% Trials: DLT rate
	% DLTs	> *θ* + 0.05	> *θ* + 0.10
(1) (0.02,0.3,0.8,0.7,400)	24.04	0	0
(2) (0.02,0.4,0.4,0.4,500)	28.78	0.9	0
(3) (0.02,0.9,0.3,0.3,300)	27.75	0.2	0
(4) (0.02,0.9,0.9,0.7,300)	16.1	0	0
(5) (0.02,0.3,0.3,0.3,300)	32.78	8.6	0.4
(6) (0.02,0.3,0.3,0.7,350)	28.83	0.7	0
(7) (0.02,0.5,0.5,0.8,400)	22.71	0	0
(8) (0.02,0.7,0.5,0.3,500)	24.76	0	0
